# The Effect of Alcoholic Extract of *Anabasis syriaca Iljin* on Biochemical and Histological Parameters in Rats

**DOI:** 10.1155/2022/6945745

**Published:** 2022-03-24

**Authors:** Suad M. Kloub, Saleem A. Banihani, Omar M. Atrooz, Wael M. Hananeh

**Affiliations:** ^1^Department of Medical Laboratory Sciences-Jordan University of Science and Technology, Irbid 22110, Jordan; ^2^Department of Biological Sciences, Faculty of Science, Mutah University, Zip Code 61710, Mutah, Jordan; ^3^Faculty of Veterinary Medicine, Department of Veterinary Pathology and Public Health, Jordan University of Science and Technology, Irbid 22110, Jordan

## Abstract

This work investigates the effect of the alcoholic extract of *Anabasis syriaca Iljin* on biochemical and histological parameters in male rats. The lethal dose (50% of the plant extract) was assessed, and three separate doses (1/10^th^, 1/15^th^, and 1/20^th^) were orally gavaged for two weeks into three study groups of animals (five rats in each group), with one group used as a control and gavaged normal saline via the same route. Blood was collected after overnight fasting, and 24 biochemical parameters were evaluated. The gross and microscopic findings were reported after the collection of specimens from the animals and processed routinely for standard histological procedures. Among all tested biochemical parameters, a significant increase was noted in fasting serum glucose (*p* ≤ 0.010), troponin (*p* ≤ 0.001), and creatine kinase (*p* ≤ 0.001), while a significant decrease was found in triglycerides (*p* ≤ 0.001) and low-density lipoprotein (*p*=0.001). On the other hand, no significant histopathological lesions were present within the examined tissues of all groups. In conclusion, ethanolic extract of *Anabasis syriaca* negatively affected the cardiac function of male rats and increased their serum glucose but reduced their serum triglycerides and low-density lipoprotein.

## 1. Introduction

Herbs have been used as the first medical treatment since the earliest days of mankind [1]. Herbal medicine was inherited from generation to the next due to the existence of all conditions necessary for its sustainability since it includes concepts and methods to protect and restore humans' health. In fact, in the current industrial world, along with the present revolution of synthetic medicine over the herbal one, the latter is still feasible, especially in the presence of new knowledge of chemical extraction. Though the main reasons of such feasibility are still the safety, the cost, and the availability of the natural products over the synthetic medicine, however, such trust is not constantly accepted.

**Figure 1:**
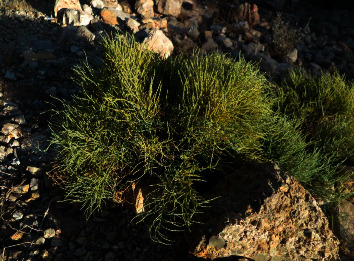
*A. syriaca* plant (https://www.plantsnap.com).

In this work, for the first time, we are adding new basic knowledge regarding the biological effects of a classified herb named *Anabasis syriaca (A. syriaca)* (Figure 1). The genus *Anabasis* belongs to the family Amaranthaceae [2]. In fact, the *Anabasis* genus comprises approximately 29 species, which are distributed from southwest Europe and North Africa to the Red Sea coast (Ethiopia) and southwest and central Asia [3].

In the present work, we hypothesized that *A. syriaca* plant may have rigorous biological effects (i.e., toxic or medicinal) in living systems, given that a previous published report revealed a toxicity of this plant for certain animals that live in the Saharan region [4]. In addition, a study conducted on certain Jordanian medicinal herbs found that *A. syriaca* has a significant antibacterial activity against *Pseudomonas aeruginosa* [5, 6]. Accordingly, we aimed to investigate the effect of alcoholic extract of *A. syriaca* on some biochemical and histological parameters in rats.

## 2. Materials and Methods

### 2.1. Preparation of Plant Extract

Fresh leaves of *A. syriaca* plant were collected from the Qastal region in Amman province. Approximately, 2 kg of the leaves were dried for two weeks at room temperature, then preserved in a plastic bag, and stored in dark for use. On the day of extraction, dried leaves were grinded into small fragments using an electric grinding machine to increase the surface area and achieve higher extraction yield. Approximately 500 g of dried *A. syriaca* plant were added to 3.125 L of ethanol (absolute, ROMIL-SpS^TM^ Super Purity Solvent, code: H314) and left in a shaking incubator with heat agitation for 48 hours at 24°C. The solution was then removed and inserted into a sonication device (Sonic Vibro Cells) for 15 minutes. Then, the entire amount of solution was filtered using filter paper inserted into a funnel-flask collector. After that, the solution was evaporated using a rotary evaporator device (model: HS-2005S-N; 395-13; Yeonghwa-dong, Jangan-gu, Korea). The yielded extract, which has sticky nature, was transported into a sterile tube and stored in dark at −20°C for use. The percent yield of extraction was estimated to be approximately 16.3%.

### 2.2. Animals

This animal experiment was conducted in the animal house at Jordan University of Science and Technology. Thirty-two apparently healthy Sprague Dawley male rats (weight: 200-260 g) were used in this experiment. The protocol of this study was read and approved by Animals Care Unit Committee at JUST. Prior the intervention, every two rats were separated together in one cage in a sterile room and left for a week to adapt in cages with provision of enough quantity of food and water.

### 2.3. Determination of LD_50_

Twelve rats were divided randomly according to weight into four groups (three rats in each group). The food was provided all day and removed at night to prepare the rats fasting to apply the LD_50_ test in the next morning. The groups were injected interperitoneally with different doses (1000 mg, 500 mg, 100 mg, and 50 mg) of *A. syriaca* extract diluted with an equal amount of normal saline (Table 1).

The rats were monitored for 24 hours to note any effect of plant extract on animals' behaviors and bodies. The results were recorded 24 hours after administration of the doses (Table 2). The LD_50_ of the *A. syriaca* plant was calculated according to the Spearman–Kärber method (LD_50_ = LD_100_ − ∑(*a* × *b*)/*n*) [7].

### 2.4. Acute Toxicity Experiment

Twenty rats were randomly divided according to weight into four groups (five animals in each group). The first group was considered as control and was gavaged with normal saline. The second group was gavaged with 22.5 mg kg^−1^ body weight, which is 1/20^th^ of LD_50_. The third group was gavaged with 30 mg kg^−1^ body weight (1/15^th^ of LD_50_), while the fourth group was gavaged with 45 mg kg^−1^ body weight (1/10^th^ of LD_50_). The rats were weighed on day 0 (before starting the experiment), day 7, and day 14. The body weight of each rat was calculated using a sensitive digital balance. In day 15, the blood was collected from all rat groups after overnight fasting and directly transferred to the laboratory.

In this experiment, it is worth mentioning that the change in the method used, which takes the rats of *A. syriaca* between injection in LD_50_ experiment and gavage in acute toxicity experiment, was to protect them from the stress that may be produced from daily injection during 14 days of acute toxicity experiment.

### 2.5. Sample Collection and Analysis

#### 2.5.1. Biochemical Specimens

On day 14 of the acute toxicity test, the animals were deprived of food at night and sacrificed at the following day using light ether anesthesia. Blood was collected in plain tubes through the cardiac puncture, just before death, for biochemical tests. The blood was directly transferred to the laboratory.

The biochemical tests (glucose, urea, creatinine, uric acid, sodium, potassium, chloride, total protein, albumin, total bilirubin, bilirubin direct, alkaline phosphatase (ALP), alanine aminotransferase (ALT), aspartate aminotransferase (AST), gamma-glutamyl transferase (GGT), creatine kinase (CK), cholesterol, triglyceride, high-density lipoprotein (HDL), low-density lipoprotein (LDL), calcium, phosphorus, and iron) were conducted on serum samples centrifuged at 3500 rpm for 15 min using the AU480 Beckman Coulter device (California, USA). The troponin test was conducted on the serum using the Access 2 Beckman Coulter device (California, USA). All tests were conducted in the Health Center Laboratories at Jordan University of Science and Technology.

#### 2.5.2. Histological Specimens

Following the blood collections, the animals were euthanized and were subjected to full necropsy. The entire organs were examined for any pathological lesions and representative tissue, and the samples were collected from different organs for histopathological examination.

The liver, kidneys, small and large intestines, spleen, pancreas, stomach, and heart were instantly fixed in 10% formalin solution for 24 h. Fixed tissues were processed routinely in an automatic tissue processor and then embedded into paraffin blocks. Three to five micrometer thick tissue sections were made and stained with hematoxylin and eosin stain for histopathological examination and evaluation.

### 2.6. Statistical Analysis

Statistical Package for Social Sciences software (SPSS version 22) was utilized to analyze data. A one-way multivariate analysis (MANOVA) was used to test mean differences between groups on several variables, and repeated measure ANOVA was used to test mean differences within groups. For all analyses, *p* values of less than 0.05 were considered significant in the assessed parameters, particularly the biochemical and the weight of rats. All continuous data were expressed in mean ± standard deviation (SD).

## 3. Results

Table 1 shows the weights of rats in each tested dose (50, 100, 500, and 1000 mg) of *A. syriaca* based on the LD_50_ experiment.

The results of LD_50_ experiment corresponding to the Spearman–Kärber equation are presented in Table 2. LD_100_ is considered as the lethal dose causing the 100% death among the tested animals. The LD_50_ of I/P injected ethanolic extract in the rats was determined and found to be 450 mg·kg^−1^.

**Figure 2:**
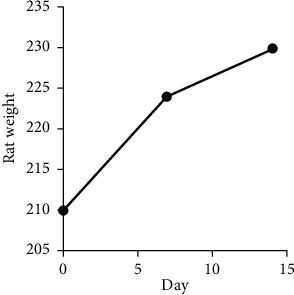
Illustrative relation of the weights of rats versus time based on the acute toxicity experiment.

Table 3 presents the changes in rats' weights monitored in the acute toxicity experiment. As shown in the table, the rats' weights were monitored at 0, 7, and 14 days following *A. syriaca* supplementation at 1/20^th^, 1/15^th^, and 1/10^th^ of LD_50_. As summarized in Figure 2, the results from toxicity experiment indicated no effect of *A. syriaca* plant administration on the average of total amount of food intake in the tested groups (2, 3, and 4) compared to the control group (1). As can be seen in the figure, there was a gradual increase in weight of rats during the experiment in all groups and this weight increase versus time was found to be statistically significant (*p* value < 0.05).

### 3.1. Effects of *A. syriaca* Extract on Biochemical Parameters

Table 4 presents the effects of *A. syriaca* extract on twenty-four biochemical parameters in Sprague Dawley male rats. Among the twenty-four tested biochemical parameters, only five (glucose, LDL, triglyceride, troponin, and CK) were found to be statistically significant (*p* value < 0.05) compared to control at all established groups.

### 3.2. Effects of *A. syriaca* on Histological Parameters on Rats

**Figure 3:**
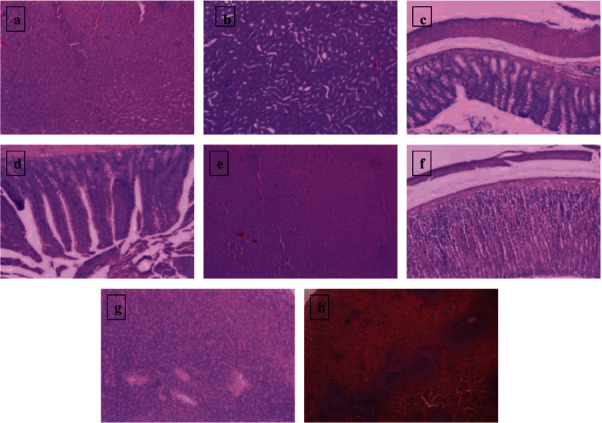
Photomicrographs of different organ systems ((a) heart, (b) kidney, (c) large intestine, (d) small intestine, (e) liver, (f) stomach, (g) pancreas, and (h) spleen) from Sprague Dawley male rats with the higher dose of *A. syriaca*. No significant histopathological lesions were found (H&E stain, 10x).

All animals in all groups survived the entire length of the experiment. No abnormal clinical signs were seen in the treatment groups in comparison with their control counterparts. No significant gross or histopathological lesions were present within the examined tissues (heart, kidney, large and small intestines, liver, stomach, pancreas, and spleen) of all groups. Histologically, the examined tissues were within normal limits (Figure 3).

## 4. Discussion

The present study, which is to our knowledge the first of its kind in this research context, was conducted to obtain the effects of *A. syriaca* plant extract on biochemical and histological parameters in male rats. Biochemical changes showed increase in serum glucose, troponin, and CK and a decrease in LDL and triglycerides. On the other hand, no histopathological lesions were found within the examined tissues (heart, kidney, large and small intestine, liver, stomach, pancreas, and spleen) of all tested groups. These results are in line with our central hypothesis.

In general, plant extracts are rich in bioactive substances that have antioxidant potential [8–10]. Such properties most of the time influence the level of lipid profile markers in biological systems [11, 12]. In most cases, this influence is shifted towards reducing these markers below than their baseline. In the present study, there was a reduction in serum levels of LDL and triglycerides following administration of ethanolic extract of *A. syriaca*, which accordingly might be due to the presence of potential antioxidant substances in this extract [13, 14]. However, the study that confirms the presence of such antioxidants in this plant will be of great importance to confirm this suggested mechanistic route.

Also, given that LDL is a synthetic macromolecule, it can be suggested that reduced levels of LDL in *A. syriaca* fed rats may be due to an increase in the catabolism or decrease in the anabolism of LDL. However, confirming this suggestion requires separate further studies that directly probe the effect of *A. syriaca* on metabolism of LDL.

On the other hand, against our expectation, fasting serum glucose was found to be higher in the *A. syriaca* extract-treated rats compared to control. In actual fact, such result is difficult to be specifically interpreted since the hyperglycemic effect of a plant extract is multifactorial annexed to a wide range of factors that may interplay with glucose homeostasis. These factors, for example, can be dietary, hormonal, or metabolic. Therefore, further experiments seem to be mandatory when there is a need to clarify the specific causative factor of such hyperglycemic effect by *A. syriaca* extract. However, in such *in vivo* system studies, stress can also be a causative factor behind such unexpected hyperglycemic effect [15].

Furthermore, cardiac markers such as troponin and CK are well established and currently used as specific and sensitive diagnostic tools to assess cardiac function [16]. Therefore, here, we asked whether such cardiac markers are altered by the effect of *A. syriaca* extract. Our results showed an elevation in both cardiac markers (approximately 13 folds for troponin and 4 folds for CK in group 4 compared with the control). These results may indicate an effect of ethanolic *A. syriaca* extract on cardiac function in the tested rats. However, specifically in this measurement, increasing the number of the tested animals shall provide more confirmation to these findings, which, in fact, is considered as one limitation of this study. In fact, elevation of cardiac markers may indicate thrombus formation, major dissection, side branch occlusion, and distal embolization, suggesting that myocardial necrosis is the main reason for the change in the cardiac markers [17, 18]. Accordingly, it can be suggested that *A. syriaca* extract may induce cardiac toxicity in rats. In actual fact, upon confirming such new finding, ethanolic *A. syriaca* extract can be used as an inducer for cardiac toxicity in the *in vivo* system studies to assess the anticardiac toxicity effect of any bioactive substance.

The results of the potassium test showed a gradual increase in the tested groups. Hyperkalaemia has been identified as one of the major problems of medical management in patients with heart failure; it has an effect on electrical conduction of the heartbeat and the imminent danger of cardiac arrest or arrhythmia. This leads to abnormalities in the conduction system and therefore may induce and provoke life-threatening rhythm disorders [19].

Acute and subacute toxicity tests are very important to be conducted because they help to evaluate the functional and morphological effects of a given substance, especially if no previous work was conducted. Lack of gross histopathological lesions in all examined groups denoted that the examined *A. syriaca* did not cause any morphological effect in the body organs. However, elevation of cardiac markers in addition to the other biochemicals in the examined treatment groups was correlated with the functional abnormalities of different organ systems made by the plant extracts. Therefore, *A. syriaca* plant may contribute to a toxic and lethal effect when used as a traditional treatment for a long period of time. However, this will remain vague as long as the vital components of this plant are unknown.

In conclusion, the results from this study reveal that *A. syriaca* plant extract reduces triglycerides and LDL basal levels in male rats, while this occurs at the expense of weakening or damaging the heart muscle and increasing the level of blood glucose.

## Figures and Tables

**Table 1:** Rats' weights in each tested dose in the LD_50_ experiment.

50 mg of extract	100 mg of extract	500 mg of extract	1000 mg of extract
220 g	207 g	253 g	221 g
252 g	250 g	202 g	258 g
217 g	214 g	211 g	203 g

**Table 2:** Results of LD_50_ experiment according to the Spearman–Kärber equation.

Dose	*N*	Died animals	*a*	*b*	*a∗b*
50 mg	3	0	__	__	__
100 mg	3	0	0	50	0
500 mg	3	2	1	400	400
1000 mg	3	3	2.5	500	1250
					1650

*N*, total number of animals in the group; *a*, average of died animals between two successive doses; *b*, differences between two successive doses; LD_100_, lethal dose causing the 100% death among the tested animals.

**Table 3:** The changes in rats' weights during the period of acute toxicity experiment.

	0 day (g)	7 days (g)	14 days (g)
*Group 1* (control)			
1	181	200	207
2	201	224	230
3	212	235	238
4	225	238	234
5	243	258	268

*Group 2* (1/20^th^ of LD_50_)			
1	184	191	192
2	200	219	230
3	218	230	231
4	224	244	248
5	231	238	223

*Group 3* (1/15^th^ of LD_50_)			
1	188	208	213
2	198	213	215
3	207	225	218
4	205	214	215
5	260	263	260

*Group 4* (1/10^th^ of LD_50_)			
1	192	210	203
2	205	200	215
3	209	219	223
4	219	227	224
5	190	210	220

**Table 4:** Values of biochemical parameters and their statistical analysis compared to control in Sprague Dawley male rats supplemented with *A. syriaca* extract at 1/20^th^, 1/15^th^, and 1/10^th^ doses of LD_50_.

Parameter	Group 1 (control)	Group 2 (1/20^th^)	Group 3 (1/15^th^)	Group 4 (1/10^th^)	*p* value
Glucose (mmol/L)	4.43 ± 0.99	5.17 ± 0.76	5.84 ± 0.89	6.34 ± 0.50	**0.010**
Cholesterol (mmol/L)	2.14 ± 0.44	2.10 ± 0.49	2.17 ± 0.40	2.23 ± 0.47	0.978
Triglyceride (mmol/L)	0.71 ± 0.06	0.53 ± 0.07	0.46 ± 0.11	0.37 ± 0.05	**≤0.001**
HDL (mmol/L)	1.22 ± 0.14	1.09 ± 0.13	1.23 ± 0.14	1.13 ± 0.20	0.441
LDL (mmol/L)	0.49 ± 0.03	0.44 ± 0.01	0.40 ± 0.05	0.37 ± 0.01	**0.001**
Urea (mmol/L)	7.64 ± 1.28	8.42 ± 1.18	7.10 ± 1.25	7.74 ± 0.52	0.344
Creatinine (mmol/L)	48.4 ± 5.69	48.2 ± 5.11	49.1 ± 3.85	51.8 ± 4.74	0.635
Uric acid (umol/L)	109 ± 48.0	91.4 ± 23.4	116 ± 41.4	104 ± 8.75	0.694
Na^+^ (mmol/L)	143 ± 0.83	141 ± 1.09	143 ± 1.22	141 ± 1.09	0.098
K^+^ (mmol/L)	4.90 ± 1.05	5.58 ± 1.09	5.44 ± 0.96	6.72 ± 0.70	0.054
Cl^−^ (mmol/L)	102 ± 0.83	103 ± 1.30	103 ± 1.67	104 ± 1.22	0.514
ALP (U/L)	120 ± 6.05	134 ± 20.8	132 ± 13.7	125 ± 26.9	0.435
ALT (U/L)	72.0 ± 16.2	90.4 ± 34.7	80.2 ± 19.5	94.6 ± 17.3	0.627
AST (U/I)	251 ± 123	398 ± 181	240 ± 85.2	330 ± 65.1	0.184
GGT (U/L)	0.86 ± 0.82	0.90 ± 0.80	0.24 ± 0.25	0.70 ± 0.47	0.365
Bilirubin total (umol/L)	12.7 ± 6.70	22.4 ± 16.7	17.1 ± 8.56	16.9 ± 5.90	0.544
Bilirubin direct (umol/L)	3.06 ± 1.61	5.34 ± 3.63	3.78 ± 1.67	4.56 ± 1.32	0.440
Total protein (g/L)	68.0 ± 2.87	70.1 ± 4.43	71.0 ± 3.36	69.6 ± 2.30	0.536
Albumin (g/L)	31.5 ± 1.13	31.9 ± 1.27	33.2 ± 1.25	33.5 ± 1.72	0.080
Troponin (ng/mL)	1.12 ± 1.39	7.74 ± 0.94	10.5 ± 1.28	14.1 ± 1.73	**≤0.001**
CK (U/L)	1290 ± 129.1	2113 ± 191.8	2845 ± 166.9	4679 ± 1022	**≤0.001**
Iron (umol/L)	33.0 ± 2.69	34.4 ± 1.65	32.2 ± 1.22	32.2 ± 4.51	0.564
Calcium (mmol/L)	2.48 ± 0.05	2.46 ± 0.01	2.48 ± 0.06	2.45 ± 0.07	0.791
Phosphorous (mmol/L)	2.45 ± 0.37	2.51 ± 0.31	2.61 ± 0.34	2.76 ± 0.37	0.564

## Data Availability

The data are available on request to the corresponding author.
